# New Insights into the Functions of Transcription Factors that Bind the RNA Polymerase Secondary Channel

**DOI:** 10.3390/biom5031195

**Published:** 2015-06-25

**Authors:** Nikolay Zenkin, Yulia Yuzenkova

**Affiliations:** Center for Bacterial Cell Biology, Institute for Cell and Molecular Biosciences, Newcastle University, Baddiley-Clark Building, Richardson Road, Newcastle upon Tyne NE2 4AX, UK

**Keywords:** RNA polymerase, secondary channel, transcription factors, DksA, Gre, transcription fidelity, transcription processivity, transcription-replication conflicts

## Abstract

Transcription elongation is regulated at several different levels, including control by various accessory transcription elongation factors. A distinct group of these factors interacts with the RNA polymerase secondary channel, an opening at the enzyme surface that leads to its active center. Despite investigation for several years, the activities and *in vivo* roles of some of these factors remain obscure. Here, we review the recent progress in understanding the functions of the secondary channel binding factors in bacteria. In particular, we highlight the surprising role of global regulator DksA in fidelity of RNA synthesis and the resolution of RNA polymerase traffic jams by the Gre factor. These findings indicate a potential link between transcription fidelity and collisions of the transcription and replication machineries.

## 1. Introduction

Transcription, the initial stage of gene expression, is intricately regulated. This regulation is vital for the coordination of transcription with other cellular processes and, ultimately, for the successful adaptation of the cell to the environment. RNA polymerase (RNAP), the enzyme of transcription, is a direct target for binding by multiple regulators that act on key RNAP domains. For example, NusA binds to the N-terminal coiled-coil element of the β' protein, the same region on the RNAP core as bound by σ^70^ [1], in order to assist the transition from initiation to elongation. NusG, its bacterial homologue RfaH, and their eukaryotic homologue Spt5 bind at the β' clamp [2,3,4] across the central cleft of RNAP, fixing the clamp domain in the closed conformation and, therefore, increasing the processivity of elongation. The transcription repair coupling factor Mfd binds near the upstream opening of the RNAP primary channel and acts as an anti-arrest factor by promoting forward translocation, which can ultimately result in displacement of stalled RNAP from the DNA template [5,6].

**Figure 1:**
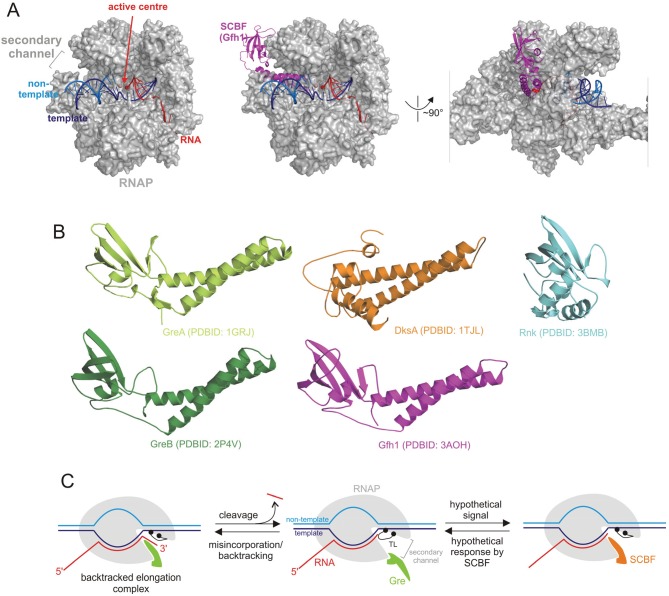
Secondary channel binding factors (SCBFs). (**A**) Structure of the transcription elongation complex [26] was superimposed with structure of the elongation complex with factor Gfh1 [27]. The RNAP core is represented as a partially transparent grey surface with nucleic acids shown as ribbons and active center Mg^2+^ ions as red spheres. Gfh1 bound in the secondary channel is shown as purple ribbons; (**B**) Aligned available structures of bacterial SCBFs. The long N-terminal coiled-coil domain protrudes through the secondary channel of RNAP (see panel B), while the C-terminal globular domain is thought to be responsible for binding to RNAP. Note that the coiled-coil domain of Rnk is shorter than those of the other SCBFs and is turned relative to the C-terminal domain; (**C**) The mode of functioning of Gre factors, and the hypothetical mode of action for other SCBFs. The Gre factor is bound to the active elongation complex but does not impose hydrolytic activity on it [28]. Upon backtracking or misincorporation, the Gre factor protrudes its coiled-coil domain through the secondary channel of RNAP, where it substitutes for the catalytic domain Trigger Loop (TL). This substitution switches off the slow TL-dependent phosphodiester bond hydrolysis and, instead, facilitates highly efficient Gre-dependent hydrolysis. After resolution of the backtracked complex through RNA cleavage, the elongation complex returns to the active conformation and the Gre factor gives way to the TL, which can now continue catalysis of RNA synthesis. The controlled switching between Gre and the TL eliminates possible interference of Gre with the RNA synthesis. It is possible that other SCBFs may act in a similar way to that of Gre by substituting the TL in the secondary channel in response to a stimuli (which remain unidentified for other SCBFs). For example, our recent results suggest that DksA may respond to binding of an incorrect NTP in the active center, and thus increase the accuracy of transcription (see text and Figure 2 for details) [29].

A distinct group of transcription factors bind at an area referred to as the secondary channel (Figure 1 and Table 1), located approximately on the opposite side of the RNAP to the binding site of the aforementioned factors. The RNAP secondary channel is a narrowing tunnel leading directly from the enzyme surface to its catalytic center (Figure 1A). This secondary channel is proposed as the main path for NTP entry into the center [7,8] and is also known to accommodate the 3' end of the RNA during polymerase backtracking [9,10]. Secondary channel-binding factors (SCBFs) are present in all three kingdoms of life—bacteria [11,12], archaea [13] and eukaryotes [14]. Below we will focus on the bacterial SCBFs; a group of factors that appear to be related to one another, but are evolutionarily unrelated to the eukaryal/archaeal SCBFs.

Bacterial SCBFs share a similar structural fold (Figure 1B). They are small proteins with a globular C-terminal domain, responsible for binding to RNAP, and a coiled-coil domain, which, at least for some of SCBFs, has been shown to be inserted through the secondary channel to modulate transcription. Two functionally important acidic residues are typically located at the tip of this domain.

The SCBFs can be roughly divided into three functional groups; the GreA/B group, the DksA group, and factors with unknown function. The precise repertoire of factors differs extensively between bacterial species, and may depend on the lifestyle and environmental niche of the bacterium. For example, at least five SCBFs have been identified in *E. coli*: GreA, GreB, DksA, TraR and Rnk. The mechanisms of *in vitro* RNAP modulation by GreA, GreB, DksA, and their *in vivo* functions, have been extensively studied [11,15,16]. TraR belongs to the DksA family, but its exact regulatory mechanism remains obscured [17]; the function of Rnk is currently unknown [18]. Most bacteria have fewer SCBFs than *E. coli*, and some bacteria with smaller genomes have GreA as their sole SCBF [19].

None of the *E. coli* SCBFs are essential for cells in normal growth conditions, although deletion of several SCBFs makes the cells sick ([20,21], and see below). At least some physiological functions of SCBFs appear to be redundant in this species. For example, overexpression of GreB compensates for the deletion of DksA [15], despite their different activities *in vitro*. The importance of SCBFs becomes apparent when a single SCBF is coded in the genome. For example, the single SCBF, GreA in both *Mycoplasma genitalium*, which has one of the smallest genome amongst bacteria [22], and its close relative *Mycoplasma pneumoniae* [23], is essential. Cells of *Streptococcus pneumoniae* (*S. pneumoniae*), which also has GreA as its sole SCBF, become extremely sick when GreA is deleted ([24], see below).

Interestingly, the genes coding for secondary channel binding factors are absent from several bacterial genera such as the cyanobacteria and several smaller groups (Aquificaceae, Dictyoglomaceae and Fusobacteriaceae) [25], suggesting that specific features of their RNAPs may compensate for the lack of SCBFs.

**Table 1:** Secondary channel binding factors discussed in this review.

Group	SCBF	Function
Cleavage factors	GreA/GreB	Resolve backtracked and misincorporated complexes via phosphodiester bond hydrolysis [11,30]. Increase clearance from some promoters [31]
DksA-like factors	DksA+ppGpp	Control isomerisation step of promoter open complexes formation on some promoters [16,32]. Increases fidelity of transcription elongation [29]
	TraR	Encoded on the conjugative plasmid. Acts similarly to DksA on initiation of transcription with no requirement for ppGpp [17]
Unknown function	Rnk	Binds RNAP but function is unknown [18]
	Gfh1/Rv3788	Inhibit catalysis by RNAP [33,34]

**Figure 2:**
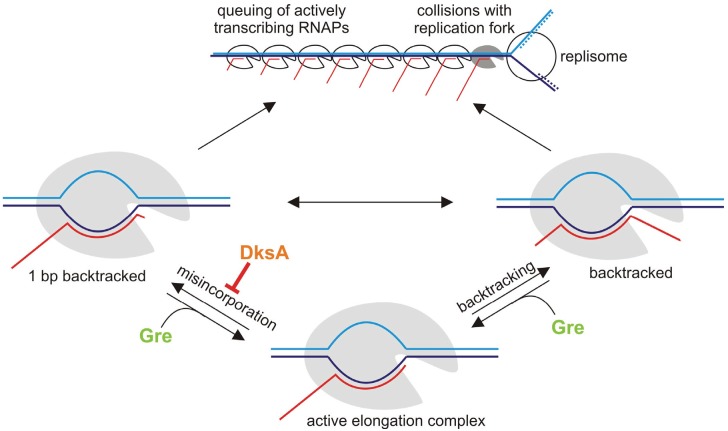
Similar roles for Gre and DksA during transcription elongation. Backtracking caused by misincorporation leads to a pause in transcription as with sequence-dependent backtracking, and may lead to the formation of RNAP traffic jams and collisions with replication forks. Gre factors resolve backtracked complexes formed by either mechanism. While DksA cannot prevent or resolve sequence-dependent backtracking, it increases accuracy of RNA synthesis and, thus, reduces the chance of misincorporation and, consequently, reduces formation of one base pair backtracked pause.

## 2. Gre Factors

GreA and GreB are transcript cleavage factors that act on backtracked elongation complexes [11,12,30]. Backtracking is a phenomenon when RNAP slides backwards along the DNA and the 3' end of the newly synthesised RNA leaves its active center (Figure 1C). While the general structure of the elongation complex (the transcription bubble, the RNA-DNA hybrid) remains unchanged during backtracking, extension of RNA becomes impossible in this conformation. However, such complexes can be resolved by the hydrolytic activity of RNAP, which cleaves the phosphodiester bond in the active center of the backtracked complex, producing a new RNA 3' end in the active center [21,35]. In the absence of a Gre factor, this hydrolytic reaction is catalyzed by a flexible domain located in the secondary channel called the Trigger Loop (TL; Figure 1C) and the two metal ions of the active center [36]. However, when bound in the secondary channel, Gre factors displace the TL from the active center [37] (Figure 1C). The displacement switches off the relatively slow TL-dependent intrinsic transcript hydrolysis, and imposes the highly efficient Gre-assisted hydrolysis. This efficiency is thought to be due to stabilisation of the second catalytic Mg^2+^ ion and an attacking water molecule by the Gre factors [37,38,39]. Notably, *E. coli* GreA has been observed in at least two conformations [33], possibly similar to those of Gfh1, which may be linked to the regulation of transcript hydrolysis (see below).

Backtracking may take place after a brief pause in transcription, caused by the thermodynamic properties of nucleic acids sequences surrounding the elongation complex [40]. In addition, misincorporation events render elongation complexes prone to backtracking by at least one bp [41,42]. In this case, the rescue from backtracking through the cleavage of the 3' end of the erroneous transcript also may be seen as a proofreading reaction. Any backtracking event causes a pause or arrest of transcription elongation, which may limit its overall rate (the average speed of RNAP along the template) or processivity (the fraction of RNAP molecules reaching the end of the gene). Accordingly, Gre factors have been shown *in vitro* to be general anti-arrest/fidelity factors [12,41,42]. However, the roles of Gre factors *in vivo* in the model bacterium *E. coli* have been non-trivial to investigate, in part due to their redundancy with additional SCBFs (see below).

*E. coli* Gre factors (GreA and GreB) appear to be not essential, possibly due to their redundancy with DksA (see below), and both can be deleted without seriously affecting cell viability [20,31]. GreB deletion has been demonstrated to have no phenotype [31]. On the background of GreB deletion, the expression levels of only a few genes were observed to change upon either GreA overproduction (105 genes were activated) or GreA deletion (19 genes were downregulated) [31]. *In vitro*, the presence of GreA decreased the fraction of abortive products from the promoters it was found to activate *in vivo.* Consequently, it was proposed that the main cellular function of GreA is to stimulate escape into productive elongation [31].

Some insights into the Gre factors’ *in vivo* functions were obtained through investigation of the GreA factor from *S. pneumoniae*. This species has a relatively small genome and encodes this single SCBF. The absence of other SCBFs makes this bacterium an ideal model with which to study the function of GreA *in vivo*. In contrast to *E. coli*, deletion of GreA in *S. pneumoniae* produced a very severe phenotype—cell growth slowed down significantly, and lysis ensued [24]. Despite the pressure on survival, no mutations that would compensate for the loss of GreA, or ease the growth defects were found.

Additionally, in contrast to *E. coli*, deletion of GreA downregulated the transcription of approximately 12.5% of the *S. pneumoniae* genome (upregulation of a similar number of genes was also observed, but was attributed to the downregulation of a general transcription repressor). As expected, *S. pneumoniae* GreA resolved backtracked complexes, and, in general, increased the processivity (success of reaching the end of the gene) of transcription *in vitro*. However, no effects on promoter escape or the other stages of transcription have been observed [24]. It was, therefore, concluded that resolution of backtracked complexes was the primary function of GreA, at least in *S. pneumoniae*. Interestingly, highly transcribed genes were affected more strongly by the deletion of GreA. Supported by stochastic simulations, this observation led to a model in which unresolved backtracked complexes may cause “traffic jams” of the RNAPs trailing behind an arrested or paused one [24].

This model contrasts with the model proposed previously for *E. coli*, which suggested that RNAP molecules cooperate during elongation in order to prevent backtracking of the leading RNAP [43]. Besides blocking gene expression of the occupied genes, queuing RNAPs would be a potent obstacle for progression of replication forks. Collisions of the transcription (especially backtracked elongation complexes) and replication machineries may be detrimental to cells if not prevented or resolved ([44,45,46]; see below). Thus, the major role of Gre factors in bacteria could involve the resolution of backtracked complexes, and the “traffic jams” caused by them, in order to ease interference between transcription and replication. However, as mentioned above, it is difficult to investigate the role of Gre in model bacteria due to redundancy in some functions with other SCBFs, such as DksA (see below).

## 3. DksA/ppGpp and TraR

DksA is a global regulator of gene expression in many bacteria [47,48]. Acting in concert with the alarmone ppGpp, DksA is involved in both positive and negative regulation of the expression of many genes during the stringent response [32,49,50]. DksA/ppGpp are best known for shutting down ribosomal transcription during starvation [16]. It has been demonstrated that DksA decreases the life-time of the open complex on the *rrnB* P1 promoter in *E. coli* [16]. Alternatively, DksA/ppGpp can directly stimulate transcription of some genes, such as those required for *de novo* amino acid biosynthesis [32]. To explain these opposing effects on transcription, it was proposed that DksA and ppGpp act synergistically by decreasing the energy barrier for open complex formation and simultaneously reducing open complex stability, which might stimulate or inhibit initiation, the outcome depending on intrinsic kinetic properties of a given promoter [32].

An alternative model for DksA and ppGpp was proposed for *Pseudomonas aeruginosa*, where it was observed that DksA, in synergy with ppGpp, actually increases the binding of RNAP to ribosomal promoters *in vitro*, presumably forming inactive complexes [51]. Moreover, in many cases where DksA and ppGpp act together (either to induce or repress transcription) the absence of ppGpp can be compensated for by overproduction of DksA, suggesting that they could act independently [52]. In some cases DksA and ppGpp behave antagonistically, for example in the regulation of the *E. coli* *fimB* promoter [53]. Recently evidence has grown to suggest that DksA can also act as an elongation factor. In support of this hypothesis, a genome-wide localization study of DksA revealed that it was distributed along transcribed sequences, presumably being associated with elongating RNAP, rather than concentrated at the promoter regions [54]. The role of DksA/ppGpp in elongation will be discussed in more detail below.

Conjugative plasmids often encode a DksA homolog, TraR, which regulates the extracytoplasmic stress response. Structural prediction of TraR indicates the presence of a single alpha helix, corresponding to half of the coiled-coil domain, which can protrude into the active center of RNAP. Unlike DksA, TraR does not require ppGpp for regulation of rRNA transcription, suggesting that it can mimic the combined activity of DksA and ppGpp on its own [17].

## 4. Factors with Unknown Functions

In contrast to GreA/B and DksA (TraR), other SCBFs, such as Gfh1 (Figure 1B) from *Deinococcus-Thermus* phylum, and Rnk (Figure 1B) from Gram-negative proteobacteria, do not appear to have any specific physiological role of their own. The best-studied example of this type of factor is Gfh1, which inhibits all the catalytic activities of RNAP—NTP addition, intrinsic hydrolysis and pyrophosphorolysis [33,55]. In a manner similar to the Gre factors, a coiled-coil domain of Gfh1 displaces the TL catalytic domain away from the active center [27] (Figure 1C), causing inhibition of transcription [37]. However, unlike the Gre factors, Gfh1 cannot hydrolyse RNA, despite the presence of conserved acidic residues at the tip of its coiled-coil domain. Interestingly, Gfh1 is active only at acidic pHs, at which it adopts GreA-like conformation. A pH increase causes inversion of the Gfh1 C-terminal domain relative to its N-terminal domain, abolishing the ability of Gfh1 to bind RNAP [33]. This pH-dependent activation mechanism suggests that Gfh1 modulates transcription under conditions where intracellular pH is lowered. Recently, a functionally similar SCBF from *Mycobacterium tuberculosis*, Rv3788, has been characterized [34]. Rv3788 competes with GreA for binding to RNAP, prevents nucleotide addition, and its two conserved acidic residues are critical for this inhibition [34]. The inhibition is also stronger at lower pH, suggesting a structural similarity with Gfh1.

The *E.coli* factor Rnk is currently assigned as regulator of nucleoside diphosphate kinase [18]. Although Rnk is structurally similar to the other SCBFs, its N-terminal coiled-coil domain is much shorter, and would not reach the RNAP active center if bound in a manner similar to Gre or Gfh1 (Figure 1B). Additionally, its C-terminal domain is turned 104° compared to that of GreA. Nevertheless, it has been shown that Rnk can bind RNAP and competes with Gre factors and DksA for this binding [18]. *E. coli* cells deleted for Rnk have no phenotype [18] in normal growth conditions.

Gfh1, Rv3788 and Rnk are all able to displace bound Gre proteins and DksA from RNAP *in vitro*, due to their higher affinity for RNAP, but the significance of this *in vivo* remains to be shown. It is possible that these factors may act as anti-Gre and/or anti-DksA factors. It is also possible that the observed inhibitory activity of some of these factors may make a significant contribution to cell maintenance in some as yet unknown conditions.

## 5. Regulation and Competition between SCBFs

As SCBFs share structural similarity, it is not surprising that they bind RNAP in a similar way. For example, DksA and GreA/GreB compete and act antagonistically on some promoters [15,56]. This situation raises the problem of how the binding of several mutually interchangeable factors, which have different activities, is regulated. In *E. coli* DksA is the most abundant SCBF in the cell and its levels do not seem to depend on growth rate [57]. GreA concentration is three times, and GreB 10 times, less than that of DksA, with Rnk concentration close to that of GreB [18,57].

One level of regulation of the SCBFs’ activities may be bound to growth conditions. For example, DksA, which acts synergistically with ppGpp, may fully manifest its function in stationary phase, when ppGpp is produced. In contrast, TraR, which does not require ppGpp, may perform its functions in the exponential phase of growth. In addition, levels of SCBF can change in response to specific conditions, for instance due to different types of stress [58,59,60,61]. However, this control mechanism may not be enough, given that GreB, the least abundant of the studied *E. coli* SCBFs, is present in the same quantity per cell as RNAP [57]. Consequently, at each moment in time, all RNAPs in the cell may be accompanied by one or the other SCBF. Furthermore, it has been observed that Gre factors may stay permanently bound to RNAP and the elongation complexes [28,62], that is, they may act as a subunit of RNAP, rather than its accessory factor (Figure 1C).

There is evidence that different SCBFs act on specific conformational or functional states of the RNAP [63], which may explain their controlled interchange. For example, *Thermus aquaticus* GreA has been shown to enter the secondary channel and substitute for the TL during misincorporation and backtracking, but not in active elongation complexes [37]. After the cleavage reaction is complete, Gre apparently withdraws from the secondary channel to allow continuation of RNA synthesis. This functional switch of Gre may involve conformational changes of the factor (Figure 1C), analogous to those recently proposed for the eukaryotic analog of GreA, TFIIS [64]. Indeed, at least two alternative conformations have been observed for GreA and its homologue Gfh1 [33]. DksA was proposed to recognise some paused elongation complexes [65] and, possibly, complexes that are about to undergo misincorporation ([29], see below). Thus, subtle conformation changes in the elongating RNAP in response to various signals may control the interchange of SCBFs in its active center. For example, bacterial species that encode DksA also possess large insertions in the TL (SI3) [63]. Alternative conformations of SI3 may provide discrimination between recruitment of DksA and the other SCBFs [63].

## 6. Misincorporation and Conflicts between Transcription and Replication

Despite different, and sometimes antagonistic activities of SCBFs, some SCBFs appear to complement each other in one important instance—in preventing conflicts between the replication and transcription machinery [66]. In rapidly growing and dividing bacterial cells replication and transcription have to proceed at the same time, and both machineries operate on the same genomic DNA [67]. As a consequence, collisions between RNAP and replication forks are highly likely to occur. Two types of collisions are possible; head to head or co-directional. Head to head collisions appear to be more detrimental to cell viability [68,69,70] since ribosomal operons and other highly transcribed genes are encoded on the leading DNA strand, and therefore transcribed codirectionally with replication fork progression [71]. However, codirectional collisions are still inevitable because the DNA polymerase moves at least 10 times faster than RNAP [72]. Resolution of both types of conflict *in vivo* requires fork restart machinery in *B. subtilis* [45]. Stalled/backtracked RNAPs present a particular challenge to replication fork progress; recently, backtracking was linked to genomic instability in *E. coli* following the observation that codirectional collisions between replication forks and backtracked RNAPs resulted in double strand breaks [44]. Inability to resolve the conflicts between the two machineries often leads to chromosomal rearrangements and deletions [73,74,75].

Gre factors and DksA have been linked with the prevention of collisions between replication forks and elongating RNAPs in *E. coli* [66,76,77]. This function appears to be redundant between GreA and DksA; deletion of DksA results in replication fork stalling in starved cells, and overexpression of GreA alleviates this effect [76]. The involvement of Gre factors in collision prevention is understandable since Gre factors can resolve backtracked elongation complexes. However, it remained unclear how the DksA factor, which does not change the catalytic properties of the RNAP active center, may reduce interference of transcription with replication. It has been hypothesised that DksA, with or without ppGpp, may prevent collisions with replication forks by displacing stalled elongation complexes, or by preventing RNAP backtracking [76]. However, neither of these activities has been observed *in vitro* [29,63,78]. In fact, DksA weakly inhibits transcription elongation *in vitro* [29,78].

As mentioned above, backtracking and pausing of transcription can be caused by misincorporation of a wrong nucleotide. Interestingly, it has recently been observed that DksA inhibits incorporation of erroneous substrates into RNA both *in vitro* and *in vivo* [29]. Further, ppGpp potentiated this function of DksA. The exact mechanism of transcriptional fidelity increase by DksA/ppGpp remains unknown. By binding in the vicinity of the active center, DksA could disfavour binding of non-cognate substrates or influence the process of TL folding, which is the major determinant of accuracy of NTP selection [79]. In addition, ppGpp, which was recently shown to bind a substantial distance from the active center, proximal to the ω subunit [80,81], may affect misincorporation allosterically by changing the overall configuration of the elongation complex. A second proposed binding site for ppGpp in the active center [82] may also be functional and play a role in reducing misincorporation. Therefore, although DksA/ppGpp cannot stop movement of RNAP backward *per se*, they can potentially prevent one of the causes of backtracking (Figure 2).

Sequence-dependent pausing seems not to involve many backtracked pauses since deletion of either Gre factor in *E. coli* does not have a significant effect on the pattern of pausing [83]. The fact that DksA/ppGpp plays a role in the resolution of conflicts between elongation complexes and replication forks suggests that misincorporation could be the major cause of the formation of backtracked complexes. Estimated frequency of misincorporation is between 10^−4^ to 10^−5^ mistakes per nucleotide, depending on the sequence [79,84,85]. In single-molecule experiments, putative misincorporation pauses were shown to be very long-lived, with a duration up to 30 min, even under saturated nucleotide conditions [86]. Consequently, if not prevented or resolved, misincorporation events may account for 40–400 strong pauses per genome (four Mbp in *E. coli*). Furthermore, taking into account the potential for paused RNAP to cause transcription “traffic jams”, relatively few misincorporation events could lead to multiple potent obstacles for the replication fork. Therefore, even the modest observed decrease in fidelity (approximately four-fold in the absence of DksA), may significantly diminish the viability of cells.

## 7. Conclusions

To summarize, GreA and DksA/ppGpp may both contribute to the prevention of conflicts between elongating RNAP and replication forks via the same pathway, but at different stages—while DksA prevents formation of misincorporated backtracked complexes, Gre resolves them (Figure 2). This model would explain how SCBFs with different activities could complement each other in the resolution of the conflicts between transcription and replication.
